# Evaluation of the effectiveness of the FOCUS ADHD App in monitoring adults with attention-deficit/hyperactivity disorder

**DOI:** 10.1192/j.eurpsy.2023.2422

**Published:** 2023-06-21

**Authors:** Luiz Roberto Carvalho, Letícia M. Haas, Gregory Zeni, Marcelo M. Victor, Stefania P. Techele, Júlia Marrone Castanho, Isabel Meneghetti Coimbra, Anthony de Freitas de Sousa, Nathalia Ceretta, Alia Garrudo, Eugenio Horacio Grevet, Luis Augusto Rohde

**Affiliations:** 1 Institute of Psychiatry, Faculty of Medicine of the São Paulo, São Paulo, Brazil; 2 Laboratorio Interdisciplinar de Neurociencias Clinicas (LiNC), Department of Psychiatry, Federal University of São Paulo, São Paulo, Brazil; 3 Attention Deficit/Hyperactivity Program and Developmental Psychiatry Program, Hospital de Clinicas de Porto Alegre, Federal University of Rio Grande do Sul, Porto Alegre, Brazil; 4 Institute of Mathematics and Statistics, University of São Paulo, São Paulo, Brazil; 5 Medical Council, UniEduK, São Paulo, Brazil; 6 Center for Research and Innovation in Mental Health, National Institute of Developmental Psychiatry, São Paulo, Brazil

**Keywords:** adherence, attention-deficit/hyperactivity disorder, digital, discount, mobile app

## Abstract

**Background:**

The current investigation assessed a) the performance of the FOCUS ADHD mobile health application (App) in increasing pharmacological treatment adherence and improving patients’ knowledge of attention-deficit/hyperactivity disorder (ADHD) and b) the impact of implementing a financial incentive for using the App (i.e., a discount on medication).

**Methods:**

In a randomized, blind, parallel-group clinical trial, 73 adults diagnosed with ADHD were allocated into three groups for 3 months: a) Pharmacological treatment as usual (TAU); b) TAU and the App (App Group); and c) TAU and the App + a commercial discount on the purchase of medication prescribed for ADHD treatment (App + Discount Group).

**Results:**

There was no significant difference in mean treatment adherence between groups, assessed as a medication possession ratio (MPR). However, the App + Discount Group exhibited greater medication intake registrations compared with the App Group during the initial phase of the trial. The financial discount also produced a 100% App adoption rate. App use did not increase ADHD knowledge, though knowledge scores were high at baseline. The usability and quality of the App were rated favorably.

**Conclusions:**

The FOCUS ADHD App achieved a high adoption rate and positive evaluations by users. Use of the App did not increase adherence to treatment as measured by MPR, but, for App users, the addition of a financial incentive to use the App produced an increase in treatment adherence in terms of medication intake registrations. The present results offer encouraging data for combining incentives with mobile digital health solutions to positively impact treatment adherence in ADHD.

## Introduction

Attention-deficit/hyperactivity disorder (ADHD) is a chronic condition characterized by inattention, hyperactivity, and impulsivity, affecting approximately 5.3% of children and adolescents globally [1]. ADHD symptoms in adults are associated with significant functional impairments, such as lower quality of life, poor academic and work performance, lower income, impairments in social life, and difficulty maintaining relationships [2]. The disorder is also associated with an increased risk of substance use, crime, incarceration, and premature death due to accidents or even suicide [2].

The ADHD diagnosis is based on clinical evaluation. As a chronic condition, patients with ADHD should undergo periodic reassessments and receive long-term monitoring [3]. Effective treatment is multi-faceted and includes psychoeducation, psychological treatment, and the use of medication [4]. Psychostimulants are the first-line pharmacological agents for ADHD treatment with high evidence of efficacy, tolerability, and safety [5]. The main treatment objective is to maximize functionality [6] by reducing symptoms, which, in turn, will reduce negative outcomes and improve quality of life [7, 8].

Despite data showing the efficacy and tolerability of available treatment interventions in clinical trials, less than 20% of adults with ADHD are diagnosed [9], only 11% of those who meet the diagnostic criteria have access to treatment [10], and only 12% of patients who start treatment persist after 12 months [11]. Both lack of treatment and low adherence to treatment are associated with significant negative outcomes to the patients and their ecosystem [12]. These factors result in expenses that threaten the viability of the entire Health System [13, 14].

### Treatment adherence

Treatment adherence is a complex phenomenon that depends on a set of interrelated domains including patient symptomatology, treatment plan, socioeconomic aspects, and the healthcare system itself [15, 16]. Though treatment adherence in ADHD remains a central challenge in patient follow-up [17], the variables affecting adherence might be preventable or amenable by relatively simple interventions [17–22].

Numerous strategies have been evaluated to improve treatment adherence in mental health. The optimization of health services design, involving specialized and/or multidisciplinary management [18] can improve patient engagement and treatment outcomes [19–21]. The implementation of patient education programs, nursing support lines, and behavioral therapy are examples of effective interventions [22]. Psychoeducational interventions aim to increase patients’ knowledge and acceptance of their health condition [23]. Providing information about ADHD, its treatment, and symptom management strategies improves treatment satisfaction and treatment adherence, and enhances self-esteem and quality of life [19, 24]. Among the studies conducted with adults, positive results were obtained regarding inattention symptoms [25, 26], self-esteem [27, 28], anxiety and depressive symptoms [27, 29], and relationships with others [28, 29]. Recently, a randomized controlled trial evaluating a smartphone-assisted psychoeducation program showed a higher homework compliance rate (93.9%) compared with a brochure-assisted psychoeducation group (66.2%) [30].

Other interventions include contingency management strategies (i.e., offering patients financial incentives to increase adherence). In substance use disorders, the use of contingency strategies has a positive impact on medication adherence, treatment attendance, and treatment goal completion, including completing homework, signing treatment plans, and reducing dysfunctional behavior [31, 32]. This strategy has also been applied to other mental health conditions [31], with positive and cost-effective results on medication adherence [32].

### Mobile digital health solutions for treatment adherence

Another promising strategy for increasing treatment adherence in mental health disorders [33], including ADHD [22], involves the use of technology-based interventions. Mobile digital health (mHealth) solutions, delivered through smartphones and other mobile devices, may help in patient education, monitoring, and treatment adherence by improving patient communication with their healthcare professionals and services [34]. In addition, the use of mHealth solutions could facilitate the tracking of treatment initiation, adherence, and efficacy [22]. Features such as reminders for medication intake, medical appointments, or prescription renewal are simple but potentially effective solutions for treatment adherence support.

To date, few studies have been conducted on ADHD evaluating the use of text messages (short message service [SMS]) to send medication reminders as an intervention to improve pharmacological treatment adherence. Two such studies evaluated the impact of SMS messages on promoting timely prescription refills in adult patients undergoing stimulant medication treatment in an experimental [35] and primary care [36] setting. Both studies showed that significantly more patients in the SMS intervention groups (68% and 81%, respectively) refilled their prescriptions in a timely manner compared to the control groups (34% and 36%, respectively). The primary care study also showed that the technological intervention led to 96% of subjects engaging with the intervention, successfully completing the 37 days of the SMS program. Finally, additional strategies using digital medication adherence solutions based on cognitive and behavioral approaches are currently being evaluated, such as gamified interfaces and/or contingency management strategies [37]. Interestingly, early evidence suggests that combining multiple strategies including gamification, dosage reminders, incentives, education, and social community interventions appears to be a promising direction for managing medication adherence [38].

### Present study: the FOCUS ADHD app

The FOCUS ADHD App was developed to help monitor patients with ADHD in the ADHD outpatient program (PRODAH) of the Federal University of Rio Grande do Sul UFRGS) at the Hospital de Clínicas de Porto Alegre (HCPA), Brazil. Through a Task Manager contained within the App, FOCUS combines multiple features and functionalities to support all aspects of the treatment trajectory for ADHD patients including treatment information registration (therapeutic plan including medication, dosage, frequency, and other clinical information), patient management support (daily pill reminders and adherence tracking, weekly ADHD symptoms evaluation, and adverse events registering), an option to add a user-customized reward system, and a robust library of psychoeducational contents. Finally, the App allows the integration of a treatment support network that can be shared with family members and health professionals, at the user’s discretion.

Although promising, many mHealth solutions lack robust scientific clinical evidence of their efficacy [39, 40]. Thus, the main aims of the current investigation were to a) assess the performance of the FOCUS ADHD mobile health App in increasing treatment adherence and improving patients’ knowledge of ADHD and b) determine the impact of implementing a financial incentive as a contingency strategy (i.e., providing a discount on the purchase of psychostimulant medication).

The main hypotheses considered for this study were a) the use of the FOCUS ADHD App during pharmacological treatment (App Group) would increase treatment adherence compared with a treatment as usual (TAU) only Control Group; b) providing a significant discount on stimulant medication (25%), combined with the use of the App (App + Discount Group) would increase medication treatment adherence compared with the TAU-only Control Group and the App Group; and c) users of the App (App Group and App + Discount Group) would obtain higher scores on an ADHD knowledge scale compared with the TAU-only Control Group.

## Methods

### Participants and recruitment

A three-arm randomized clinical trial was conducted to monitor adult participants with ADHD for 3 months. The participants were recruited through a public call led by the PRODAH-Adult division between June 2021 and October 2022.

The participants were evaluated according to the following inclusion criteria: a) fulfill diagnostic criteria for ADHD according to the DSM-5, b) age range between 18 and 45 years of age, c) own a smartphone, d) have a clinical ASRS score at diagnosis of ≥24, e) have at least a high school diploma, and f) accept the use of pharmacological treatment for ADHD. The exclusion criteria for the study were a) participants with any unstable or chronic clinical disease without adequate treatment, as hypertension, heart, kidney, or liver diseases; b) the presence of any significant neurological disease (e.g., delirium, dementia, epilepsy, ALS, TAND, multiple sclerosis, and stroke); and c) the presence of unstable psychiatric comorbidities requiring immediate treatment such as depression with risk of suicide, substance abuse/dependence, anxiety, bipolar disorder, and current or past history of psychosis.

Prior to the trial, participants were evaluated by a clinically experienced psychiatrist using the Structured Clinical Interview for DSM-IV and the Mini International Neuropsychiatric Interview. Sociodemographic and medical history information was also collected. Participants answered the ASRS-18 questionnaire version validated in Brazil [41] for the evaluation of ADHD symptoms.

**Figure 1.**
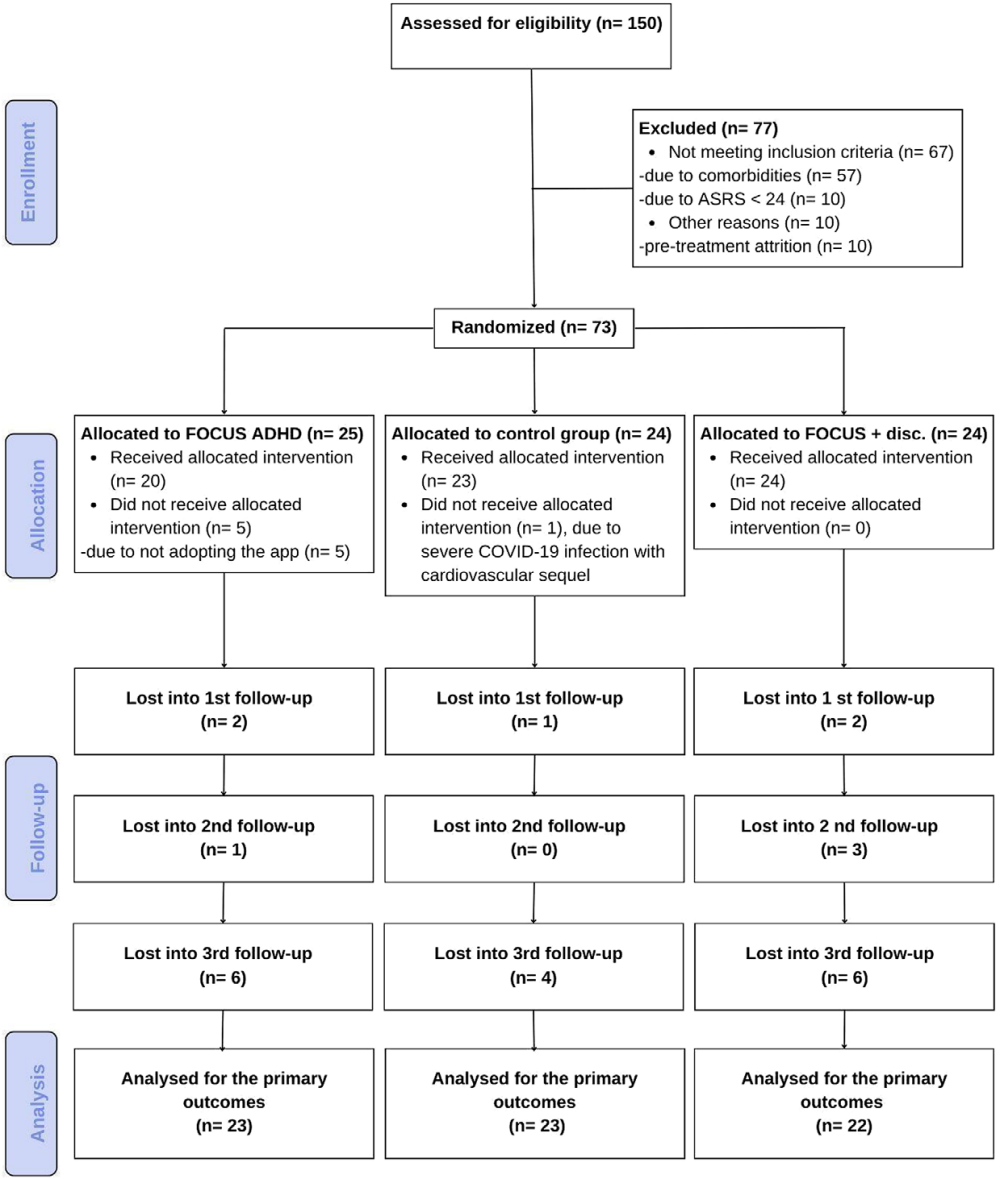
CONSORT flowchart.

Before starting the trial, participants were given a Baseline clinical evaluation by a psychiatrist with extensive experience with ADHD who confirmed the diagnosis based on a clinical interview and the application of the Portuguese version of the ADHD module of the K-SADS-E adapted for adults [42]. Participants needed to meet all DSM-5 criteria for ADHD in adults and have a minimum clinical ASRS score of 24 at baseline (see the study flowchart in Figure 1, and the protocol workflow in Supplementary Figure 1).

### Sample description/characteristics

The eligible participants were then randomized into three groups by an outside member of the research team who did not participate in their follow-up. Participants randomized to the first group followed pharmacological treatment as usual (TAU-only Control Group). TAU consisted of clinical pharmacotherapy administered by a psychiatrist experienced in the treatment of adult ADHD. The second group, in addition to TAU, used the FOCUS ADHD App (App Group). Participants in the third group followed TAU and used the FOCUS ADHD App (like the App Group), but also received a discount on their medication purchase, conditioned on participating in at least 80% of the planned monthly medication intakes as established by the treatment plan registered in the App (App + Discount Group).

Clinical follow-up assessments were conducted by the same psychiatrist who conducted the Baseline clinical evaluation after 4 weeks (second clinical evaluation) and 8 weeks (third clinical evaluation). The clinician performed treatment adjustments, refilled the prescriptions, and evaluated ADHD symptoms using the ASRS scale. A final follow-up was also performed at 12 weeks using electronic self-assessment questionnaires sent to participants.

Treatment adherence was monitored through weekly contacts by research assistants with all participants. The instrument used to evaluate adherence to pharmacological treatment was the medication possession rate (MPR) – defined as the average percentage of time (days covered by the medication dosage) a patient has access to treatment according to their treatment plan between two prescriptions. A cutoff of 80% (or a mean MPR of <0.8) was used to indicate low treatment adherence [22].

The knowledge assessment was performed based on an adapted questionnaire [43] to identify participants’ awareness and understanding of ADHD. This consisted of an 11-item Likert Scale from 1 (totally disagree) to 5 (totally agree), where a total score equal to or higher than 7 points was used as a cutoff for a “good” understanding of ADHD consistent with previous investigations [43]. The participants’ knowledge of ADHD was assessed at the beginning of the study at the Baseline clinical evaluation and at the end of the follow-up period (12 weeks). During the protocol duration, the App users received psychoeducational multimedia ADHD contents twice a week that could be accessed directly in the App or at the FOCUS ADHD website. The publications included short videos and texts covering different facets of adult ADHD-related information such as what the disorder is, its potential causes, clinical presentations and comorbidities, and strategies to deal with ADHD. Users were sent push notifications and messages to inform them about content updates.

Participants assigned to the two FOCUS ADHD App groups were given instructions concerning App download and registration by the study coordinator. They were also informed about the App functionalities such as the pill reminders, weekly symptoms assessment, the option to invite a support network, and the twice-a-week psychoeducation contents that would be published during the protocol duration. The App + Discount Group was also informed about the incentive eligibility criteria, namely, to register 80% of their medication intakes on the App according to the treatment plan registered on the tool by the user. Once a week, the App database was analyzed to check participants’ adherence to determine eligibility for the discount at the end of the first and second months. If a participant was eligible, instructions were provided explaining how to access a 25% discount from a partner Pharmacy Chain.

Participant ratings of App quality were evaluated through the User Version of the Mobile Application Rating Scale (uMARS) [44] that was adapted to Portuguese. The uMARS is a 20-item Likert Scale from 1 (low) to 5 (high) that includes four objective quality subscales – Engagement, Functionality, Esthetics, and Information Quality – and one subjective Quality subscale [44]. The total score, ranging from 1 to 5, is calculated as the mean of the four objective subscales. A score above 3 or higher indicates satisfactory app quality. The participants rated the App using the uMARS scale, after interacting with the App for 2 weeks. The questionnaire was sent again after 12 weeks of usage (i.e., at study completion).

### Statistical analysis

The final analyzed sample comprised all participants with available data in at least one of the primary outcomes, namely, treatment adherence, knowledge of ADHD, or quality of the App.

All analyses were performed using JAMOVI software (Version 2.3) [45]. Mean, standard deviation, and absolute and relative frequencies were used to describe the sample. Normality tests and distribution techniques were used to explore quantitative distribution variables. Categorical data were analyzed using *X*^2^ and Fisher exact tests. The *t*-Student or *U*-Mann–Whitney test was applied to compare the effect of the discount incentive on the App + Discount Group compared to the App Group. An ANOVA or Kruskal Wallis test was used to compare the three groups, and post hoc analyses with Holm’s correction were performed for multiple pairwise comparisons. To study the between-subjects factor (Group) and within-subject factor (Time), we performed a repeated measure two-way ANOVA including the interaction term. Finally, we used Spearman’s correlation analysis to evaluate the association between quantitative variables. The threshold for statistically significant effects was set at 5% (*p* ≤ 0.05).

## Results

Seventy-three participants (Mean age = 35 ± 7 years) were randomized, of which 68 were included in the analysis (see Figure 1). A statistically significant difference was found for age between groups: App Group, 39 ± 6 years; App + Discount Group, 34 ± 6 years; and TAU Group, 34 ± 7 years. The average family income was 6.600 reais (SD = 9.300). Although there was a difference between the App Group (8.890 reais, SD = 13.810), App + Discount group (5.300 reais, SD = 3.380), and the TAU Control Group (5.310 reais, SD = 6.130), this difference was not statistically significant, given a large amount of variability within groups (*p* = 0.787). Almost half of the participants were female (49.3%), and more than 80% of the participants indicated their race as white. Most participants were single (64.3%), and 62% had completed higher education. All other sample characteristics and sociodemographic factors did not significantly differ among groups (Supplementary Table 1).

Almost all participants had symptoms of ADHD before 12 years of age (98.6%). The most prevalent ADHD presentations were combined (54.8%) and inattentive (38.4%). The most commonly identified comorbid disorder was past Major Depressive Disorder (65.8%). Social Anxiety (12.3%) and Generalized Anxiety Disorder (8.3%) were also prevalent. The three experimental groups did not significantly differ in the prevalence of ADHD presentation or comorbid conditions (see Supplementary Table 2 for all details).

### Symptom response to medication

**Figure 2.**
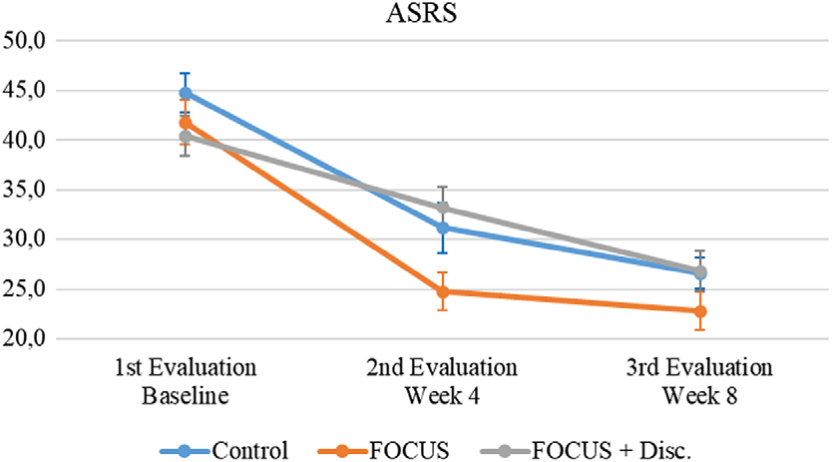
Attention-deficit/hyperactivity disorder symptoms evolution. Note. Mean (IC – 95%).

Figure 2 illustrates that clinician-administered ASRS scale scores in all participants had a significant decrease in ADHD symptoms (i.e., improvement) over Time (*F* = 71.24, *p* < 0.001, ES = 0.341). There was no statistically significant main effect of Group (*F* = 2.55, *p* = 0.087, ES = 0.027) and no significant Group X Time interaction (*F* = 1.82, *p* = 0.129, ES = 0.017).

### Adherence to pharmacological treatment of ADHD

**Figure 3.**
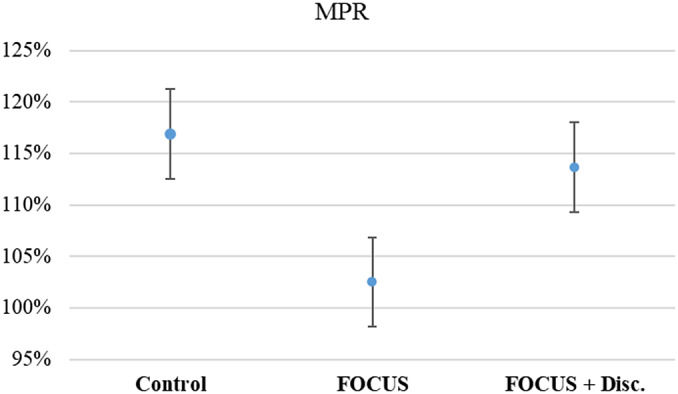
Average medication possession rate. Note. Mean (IC – 95%).

All groups had a mean MPR above 100%, and there was no statistically significant difference between groups (KW = 1.00, *p* = 0.606, ES = 0.0176) (see Figure 3). Even when establishing an 80% cutoff, there was no significant association between the pharmacological treatment adherence and groups (*X*^2^ = 0.96, *p* = 0.677).

### Participants’ knowledge of ADHD

**Figure 4.**
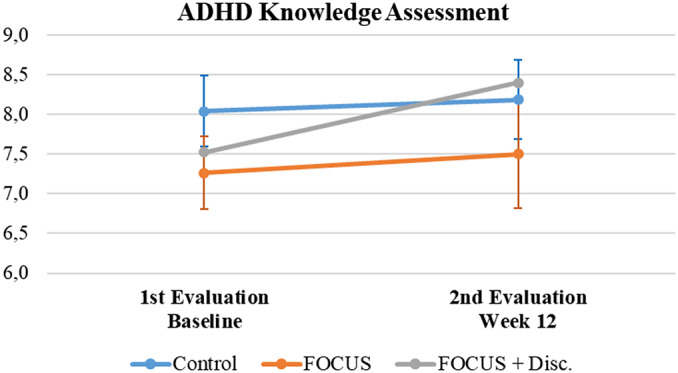
Attention-deficit/hyperactivity disorder knowledge assessment summary. Note. Mean (IC – 95%).

When evaluating the results regarding the ADHD knowledge assessment, all groups initially presented with high scores – above the cutoff score used to classify a “good” ADHD understanding/knowledge. Even though participants had a marginal increase in their scores, this was not statistically significant (*F* = 1.22, *p* = 0.275, ES = 0.007) and the result was independent of randomized group status (Interaction effect: *F* = 0.45, *p* = 0.641, ES = 0.005; Group effect: *F* = 1, *p* = 0.376, ES = 0.034) (see Figure 4).

### Adoption, engagement, retention, and the quality of the FOCUS ADHD App

Forty-nine participants were eligible to use the FOCUS ADHD App (see Figure 1).

#### Adoption

Among these 49, 5 (10.2%) did not download the App. All five participants that did not download the App were from the App Group. Thus, the adoption of the App for this group (75%) was significantly lower compared with the App + Discount Group (100%) (*X*^2^ = 5.35, *p* = 0.05).

#### Engagement

**Figure 5.**
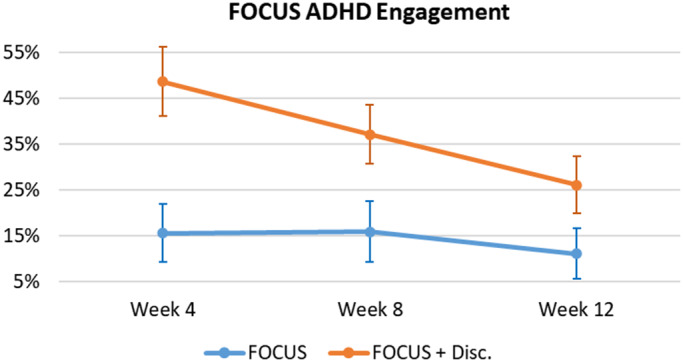
FOCUS ADHD – users’ engagement. Note. Mean (IC – 95%).

Engagement was defined as the number of times (as a %) that users registered their medication intake into the App (see Figure 5). There was a significant effect of Group as the App + Discount Group had significantly higher engagement compared with the App Group (*F* = 7.19, *p* = 0.010, ES = 0.123); and a significant effect of Time, as App engagement significantly decreased over time (*F* = 10.17, *p* < 0.001, ES = 0.028). An observed significant Time X Group interaction (*F* = 4.68, *p* = 0.012, ES = 0.013) demonstrates that the increased medication intake registrations exhibited by the App + Discount Group were significantly greater early in the trial, returning to levels similar to the App Group by the third clinical evaluation (Week 12).

#### Retention

Retention is defined as user engagement (i.e., the percentage of subjects who registered their medication intake at least 80% of the time) being consistent throughout the study (i.e., at 4 and 8 weeks). Only four participants, all from the App + Discount Group (16.7%), fulfilled this retention criterion throughout the entire study duration.

#### Quality

The FOCUS ADHD App received a positive quality evaluation by 25 study participants obtaining a total uMARS score of 3.45 (SD 0.63) out of 5 (see Supplementary Table 3). There was a trend toward greater ratings of usability in the App + Discount Group (Mean = 3.62; SD = 0.61) compared to the App Group (Mean = 3.16; SD = 0.59), but this effect was not significant (*U* = 40, *p* = 0.074, ES = 0.444). Analyzing the results from the uMARS subscales, the “Information Quality” rating received the highest mean score at 3.84 (SD 0.85), followed by Functionality at 3.67 (SD 0.88) and Esthetics at 3.49 (SD 0.66). The only rating variable with a score lower than 3 was Engagement at 2.89 (SD 0.62). Comparing the differences between the groups, the only significantly different subscale score was “Information Quality” in favor of the App + Discount Group (*U* = 28.5, *p* = 0.043, ES = 0.525).

## Discussion

The present study assessed the effect of using digital technologies for enhancing pharmacological treatment adherence in ADHD. We found no significant difference in treatment adherence (as measured by MPR) between participants receiving pharmacological TAU compared to participants who received TAU combined with the use of the FOCUS ADHD mobile health App. Though not consistent with previous studies showing a positive effect of mHealth solutions on treatment adherence [34–37], we note that these studies used different interventions and methodologies. However, our measure of App engagement (i.e., participant registration of medication intake) provides some initial evidence of the positive effect of combining a financial incentive with the use of the FOCUS ADHD App on treatment adherence.

### Treatment adherence and App engagement

Though no effect on adherence was observed, it is important to highlight that all groups exhibited an MPR above 100% in our study. These values are much higher than the established cutoff value used to classify participants as adherent and non-adherent in previous studies [22]. One possibility is that our study parameters, including weekly calls to participants from research assistants as well as scheduled assessments with the study psychiatrist, might have led to an inflated MPR in all groups. Furthermore, MPR does not guarantee that the prescribed and dispensed medication was taken in the correct manner by the patient. Since there is no “gold standard” for measuring adherence, the use of combinations of measurement tools is recommended for a more accurate assessment [16, 22, 32].

Regarding the FOCUS ADHD App, we observed a high adoption rate, especially by those receiving a reinforcement for using the App (100% for the App + Discount vs. 75% for the App Group). User engagement has proved to be a key element in determining the effectiveness of digital interventions [46–48], and here we show that financial incentives improved engagement. Indeed, registrations of medication intake were significantly higher in the App + Discount Group compared with the App Group, especially during the early phase of the trial (see Figure 5). This was expected since those in the App + Discount Group had a greater incentive for registering medication intake during the first two study assessment periods (i.e., between Baseline and the first month, and then between the first and second months) because only after these two time periods were they eligible for the discount. Since they would not receive the discount after the third assessment, this motivation decreased between the second and the third assessments, and their engagement decreased to levels similar to the non-reinforced App Group.

Related to the complexities of using MPR as a measure of adherence, it is interesting to consider that our participants’ engagement with the App and their registration of medication intake could be considered a reasonable measure of adherence. The naturalistic nature of the present study prevented us from documenting that these registrations were associated with actual medication intake. That said, the fact that our participants made an effort to get onto the App and registered an intake and confirmed to our research assistants (via weekly contacts) that these registrations were associated with actual medication intakes, provides reasonable evidence that they took the medication. This suggests initial evidence that a financial incentive paired with the FOCUS ADHD App increased medication adherence, at least initially.

In terms of retention of App use, no participant continued to register medication intake more than 80% of the time during the entire study in the App Group, while four participants in the App + Discount Group (16.7%) fulfilled this criterion. Though these numbers are low, they are consistent with the available mHealth literature [46–48], highlighting that when evaluating digital health solutions, user engagement and adherence to treatment in chronic disorders like ADHD remain central challenges in the health field.

When evaluating ADHD knowledge assessment, no significant differences emerged among groups. However, the average baseline scores for the three groups were already above the adopted cutoff for indicating a good understanding of ADHD, which may have created a ceiling effect where only a small improvement could be observed in all groups. Creating a more interactive educational experience by customizing the available psychoeducational contents with specific patient characteristics or needs could improve the FOCUS ADHD App.

When we analyzed the FOCUS ADHD App performance based on the uMARS questionnaire, the total average score of the evaluations from both groups of App users was above 3, a cutoff that has been utilized in previous studies as evidence of a positive evaluation of App quality [49–52]. In particular, the information quality of the App was recognized as its greatest strength, which may be related to the video and text psychoeducational contents. That said, digital health design requires a remarkable speed of innovation where developers must constantly update their products to align with user demands for novel design features that encourage user engagement. Continuing with this approach might increase both adoption and continued engagement with the App during treatment [53].

The literature also highlights the importance of designing and implementing personalized approaches to promote users’ engagement with digital mental health solutions [54]. The FOCUS ADHD App engages the user according to their specific registered treatment plan. In the present study, we did not personalize, for example, different psychoeducational contents based on the user’s profile and preferences, or present information using friendly interfaces, such as AI chatbots. These new approaches might increase users’ intrinsic motivation to engage within the App.

As clinical trials using digital technology continue, it will be important to consider the conditions for optimal implementation in clinical practice [55, 56]. Specifically, it will be critical to consider the involvement and engagement of healthcare personnel, as a care line in the deployment of such technology [57–61]. These changes will facilitate technology development, implementation, and assessment of mental health.

There are some limitations to consider when evaluating the present results. The sample size was relatively small, and the protocol did not have a digital control group. There were income differences among study groups that were non-significant probably due to the large amount of variability within groups. Although this difference was not statistically significant, income could affect the salience of financial incentives.

The current findings demonstrated that the FOCUS ADHD App obtained a high adoption rate and received positive evaluations from its users. In the present study, the use of the App did not increase treatment adherence as measured by MPR. That said, for those using the App, evidence of increased medication intake registrations in the group that received a discount on their medication offers initial evidence supporting the utility of combining financial incentives with mHealth solutions to increase treatment adherence. These data, together with prior evidence showing that App use can increase treatment adherence [34–37], call for future pragmatic trials designed to evaluate the effectiveness of the current proposed mHealth solution in real-life routine practice conditions.

## Supplementary material

For supplementary material accompanying this paper visit http://doi.org/10.1192/j.eurpsy.2023.2422. click here to view supplementary material
