# Supplementary material for: Evaluation of the effectiveness of the FOCUS ADHD App in monitoring adults with attention-deficit/hyperactivity disorder
Source: Eur Psychiatry. 2023 Jun 21;66(1):e53. doi: 10.1192/j.eurpsy.2023.2422 (PMC10377453; doi:10.1192/j.eurpsy.2023.2422)
Supplement: Supplementary file 1 [file S0924933823024227sup001.docx]

**Supplementary Information**

**Figure 1 - Protocol Workflow**


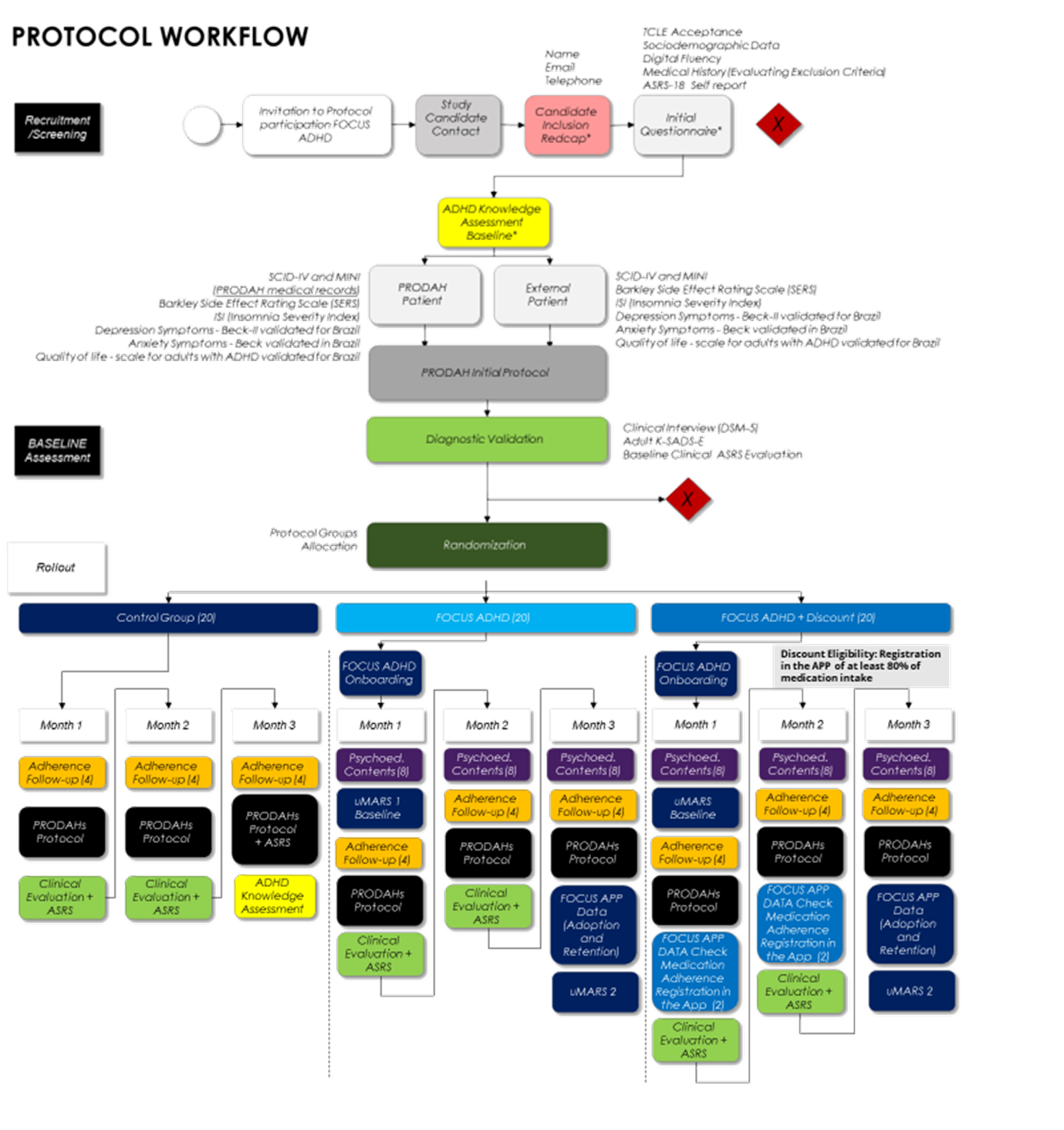


**Table 1. Sample Characteristics and Sociodemographic Factors**

|  | | **Control** | | | **Focus** | | | **Focus + Discount** | | | **Total** | | |  |  |
| --- | --- | --- | --- | --- | --- | --- | --- | --- | --- | --- | --- | --- | --- | --- | --- |
|  |  | **N** | **M** | **SD** | **N** | **M** | **SD** | **N** | **M** | **SD** | **N** | **M** | **SD** | **Stat** | **p-value** |
| **Age^1^** | | 23 | 34.2 | 7.66 | 22 | 39.2 | 6.47 | 24 | 34.6 | 6.11 | 69 | 35.9 | 7.04 | 3.9 | 0.029 |
| **Family_Income^2^** | | 20 | 5.31 | 6.13 | 24 | 8.89 | 13.81 | 21 | 5.33 | 3.38 | 65 | 6.64 | 9.29 | 0.5 | 0.787 |
|  |  |  |  | **% col** |  |  | **% col** |  |  | **% col** |  |  | **% col** |  |  |
|  | **Female** | 13 |  | 54.2% | 13 |  | 52.0% | 10 |  | 41.7% | 36 |  | 49.3% |  |  |
| **Race^4^** | **Non-White** | 4 |  | 17.4% | 4 |  | 16.7% | 6 |  | 25.0% | 14 |  | 19.7% | 0.6 | 0.807 |
|  | **White** | 19 |  | 82.6% | 20 |  | 83.3% | 18 |  | 75.0% | 57 |  | 80.3% |  |  |
| **Civil Status ^3^** | **Single/Divorced** | 14 |  | 63.6% | 15 |  | 62.5% | 16 |  | 66.7% | 45 |  | 64.3% | 0.10 | 0.953 |
|  | **Married** | 8 |  | 36.4% | 9 |  | 37.5% | 8 |  | 33.3% | 25 |  | 35.7% |  |  |
| **Education^4^** | **Postgraduate** | 5 |  | 21.7% | 9 |  | 37.5% | 10 |  | 41.7% | 24 |  | 33.8% | 5.7 | 0.491 |
|  | **University** | 9 |  | 39.1% | 6 |  | 25.0% | 5 |  | 20.8% | 20 |  | 28.2% |  |  |
|  | **High School** | 2 |  | 8.7% | 1 |  | 4.2% | 4 |  | 16.7% | 7 |  | 9.9% |  |  |
|  | **University Incomplete** | 7 |  | 30.4% | 8 |  | 33.3% | 5 |  | 20.8% | 20 |  | 28.2% |  |  |
| **Employment^3^** | **No** | 7 |  | 30.4% | 6 |  | 25.0% | 4 |  | 16.7% | 17 |  | 23.9% | 1.2 | 0.537 |
|  | **Yes** | 16 |  | 69.6% | 18 |  | 75.0% | 20 |  | 83.3% | 54 |  | 76.1% |  |  |
| *1 ANOVA; 2 Kruskal Wallis; 3 X2; 4 Fisher's exact test* | | | | | | | | | | | | | | | |

**Table 2. Comorbidities and ADHD Type**

|  |  | **Control** | | | **Focus** | | | **Focus + Disc.** | | | **Total** | |  |  |
| --- | --- | --- | --- | --- | --- | --- | --- | --- | --- | --- | --- | --- | --- | --- |
|  |  | **N** | **% lin** | **% col** | **N** | **% lin** | **% col** | **N** | **% lin** | **% col** | **N** | **% col** | **X²** | **p-val** |
| **Current major depressive disorder¹** | *Absent* | 23 | 31.9% | 95.8% | 25 | 34.7% | 100.0% | 24 | 33.3% | 100.0% | 72 | 98.6% | 2.07 | 0.658 |
|  | *Present* | 1 | 100.0% | 4.2% | 0 | 0.0% | 0.0% | 0 | 0.0% | 0.0% | 1 | 1.4% |  |  |
| **Past major depressive disorder** | *Absent* | 8 | 32.0% | 33.3% | 8 | 32.0% | 32.0% | 9 | 36.0% | 37.5% | 25 | 34.2% | 0.18 | 0.915 |
|  | *Present* | 16 | 33.3% | 66.7% | 17 | 35.4% | 68.0% | 15 | 31.3% | 62.5% | 48 | 65.8% |  |  |
| **Current mania episode** | *Absent* | 24 | 32.9% | 100.0% | 25 | 34.2% | 100.0% | 24 | 32.9% | 100.0% | 73 | 100.0% | - | - |
| **Past mania episode¹** | *Absent* | 24 | 33.3% | 100.0% | 25 | 34.7% | 100.0% | 23 | 31.9% | 95.8% | 72 | 98.6% | 2.07 | 0.658 |
|  | *Present* | 0 | 0.0% | 0.0% | 0 | 0.0% | 0.0% | 1 | 100.0% | 4.2% | 1 | 1.4% |  |  |
| **Current hypomanic episode** | *Absent* | 24 | 32.9% | 100.0% | 25 | 34.2% | 100.0% | 24 | 32.9% | 100.0% | 73 | 100.0% | - | - |
| **Past hypomanic episode¹** | *Absent* | 23 | 32.4% | 95.8% | 24 | 33.8% | 96.0% | 24 | 33.8% | 100.0% | 71 | 97.3% | 1.01 | ~1 |
|  | *Present* | 1 | 50.0% | 4.2% | 1 | 50.0% | 4.0% | 0 | 0.0% | 0.0% | 2 | 2.7% |  |  |
| **Persistent Depression** | *Absent* | 24 | 32.9% | 100.0% | 25 | 34.2% | 100.0% | 24 | 32.9% | 100.0% | 73 | 100.0% | - | - |
| **Substance induced depressive disorder** | *Absent* | 24 | 32.9% | 100.0% | 25 | 34.2% | 100.0% | 24 | 32.9% | 100.0% | 73 | 100.0% | - | - |
| **Other bipolar disorder¹** | *Absent* | 23 | 32.4% | 95.8% | 25 | 35.2% | 100.0% | 23 | 32.4% | 95.8% | 71 | 97.3% | 1.07 | 0.543 |
|  | *Present* | 1 | 50.0% | 4.2% | 0 | 0.0% | 0.0% | 1 | 50.0% | 4.2% | 2 | 2.7% |  |  |
| **Substance Use Disorder¹** | *Absent* | 23 | 33.8% | 95.8% | 24 | 35.3% | 96.0% | 21 | 30.9% | 87.5% | 68 | 93.2% | 1.79 | 0.520 |
|  | *Present* | 1 | 20.0% | 4.2% | 1 | 20.0% | 4.0% | 3 | 60.0% | 12.5% | 5 | 6.8% |  |  |
| **Panic disorder¹** | *Absent* | 24 | 33.3% | 100.0% | 25 | 34.7% | 100.0% | 23 | 31.9% | 95.8% | 72 | 98.6% | 2.07 | 0.658 |
|  | *Present* | 0 | 0.0% | 0.0% | 0 | 0.0% | 0.0% | 1 | 100.0% | 4.2% | 1 | 1.4% |  |  |
| **Agoraphobia** | *Absent* | 24 | 32.9% | 100.0% | 25 | 34.2% | 100.0% | 24 | 32.9% | 100.0% | 73 | 100.0% | - | - |
| **Social phobia¹** | *Absent* | 21 | 32.8% | 87.5% | 22 | 34.4% | 88.0% | 21 | 32.8% | 87.5% | 64 | 87.7% | ~0 | ~1 |
|  | *Present* | 3 | 33.3% | 12.5% | 3 | 33.3% | 12.0% | 3 | 33.3% | 12.5% | 9 | 12.3% |  |  |
| **Generalized anxiety disorder¹** | *Absent* | 22 | 33.3% | 91.7% | 22 | 33.3% | 91.7% | 22 | 33.3% | 91.7% | 66 | 91.7% | ~0 | ~1 |
|  | *Present* | 2 | 33.3% | 8.3% | 2 | 33.3% | 8.3% | 2 | 33.3% | 8.3% | 6 | 8.3% |  |  |
| **ADHD symptoms before 12 years old¹** | *Yes* | 23 | 31.9% | 95.8% | 25 | 34.7% | 100.0% | 24 | 33.3% | 100.0% | 72 | 98.6% | 2.07 | 0.658 |
|  | *No* | 1 | 100.0% | 4.2% | 0 | 0.0% | 0.0% | 0 | 0.0% | 0.0% | 1 | 1.4% |  |  |
| **ADHD presentation¹** | *Inattentive* | 8 | 28.6% | 33.3% | 11 | 39.3% | 44.0% | 9 | 32.1% | 37.5% | 28 | 38.4% | 0.91 | 0.926 |
|  | *Hyperactive* | 2 | 40.0% | 8.3% | 1 | 20.0% | 4.0% | 2 | 40.0% | 8.3% | 5 | 6.8% |  |  |
|  | *Combined* | 14 | 35.0% | 58.3% | 13 | 32.5% | 52.0% | 13 | 32.5% | 54.2% | 40 | 54.8% |  |  |
| **Actual pharmachotherapy (Treatment as Usual - TAU)** | *Yes* | 24 | 32.9% | 100.0% | 25 | 34.2% | 100.0% | 24 | 32.9% | 100.0% | 73 | 100.0% | - | - |
| **Actual psychotherapy** | *Yes* | 12 | 40.0% | 50.0% | 8 | 26.7% | 32.0% | 10 | 33.3% | 41.7% | 30 | 41.1% | 1.64 | 0.440 |
|  | *No* | 12 | 27.9% | 50.0% | 17 | 39.5% | 68.0% | 14 | 32.6% | 58.3% | 43 | 58.9% |  |  |
| ¹ *Fisher's exact test* | | | | | | | | | | | | | | |

**Table 3. Mean uMARS Score Evaluation**

|  | **App** | | | **App + Discount** | | | **Total Focus** | | |  |  |
| --- | --- | --- | --- | --- | --- | --- | --- | --- | --- | --- | --- |
|  | **N** | **M** | **DP** | **N** | **M** | **DP** | **N** | **M** | **DP** | **Stat** | **p** |
| **uMARS** | 9 | 3.16 | 0.59 | 16 | 3.62 | 0.61 | 25 | 3.45 | 0.63 | 40.00 | 0.074 |
| **Engagement** | 9 | 2.64 | 0.57 | 16 | 3.02 | 0.62 | 25 | 2.89 | 0.62 | 44.50 | 0.125 |
| **Functionality** | 8 | 3.54 | 0.88 | 16 | 3.74 | 0.89 | 24 | 3.67 | 0.88 | 58.50 | 0.759 |
| **Aesthetics** | 8 | 3.20 | 0.59 | 16 | 3.63 | 0.66 | 24 | 3.49 | 0.66 | 37.50 | 0.108 |
| **Information** | 8 | 3.41 | 0.92 | 15 | 4.08 | 0.73 | 23 | 3.84 | 0.85 | 28.50 | 0.043 |
